# Molecular epidemiology of Usher syndrome in Italy

**Published:** 2011-06-22

**Authors:** Diego Vozzi, Anu Aaspõllu, Emmanouil Athanasakis, Anna Berto, Antonella Fabretto, Danilo Licastro, Maigi Külm, Francesco Testa, Patrizia Trevisi, Marju Vahter, Carmela Ziviello, Alessandro Martini, Francesca Simonelli, Sandro Banfi, Paolo Gasparini

**Affiliations:** 1 Medical Genetics, Department of Reproductive Sciences, Development and Public Health, IRCCS-Burlo Garofolo Children Hospital, University of Trieste, Trieste, 34100 Italy; 2 Asper Biotech, Vaksali 17a, 50410 Tartu, Estonia; 3 Audiology, University of Ferrara, Italy; 4 CBM scrl - Genomics, Area Science Park, Basovizza, Trieste, Italy; 5 Department of Ophthalmology, Second University of Naples, Napoli, Italy; 6 Medical Genetics, Department of General Pathology, Second University of Naples, Naples, Italy; 7 Otosurgery-ENT Dept., University of Padova, Italy

## Abstract

**Purpose:**

Usher syndrome is an autosomal recessive disorder characterized by hearing and vision loss. Usher syndrome is divided into three clinical subclasses (type 1, type 2, and type 3), which differ in terms of the severity and progression of hearing loss and the presence or absence of vestibular symptoms. Usher syndrome is defined by significant genetic heterogeneity, with at least 12 distinct loci described and 9 genes identified. This study aims to provide a molecular epidemiology report of Usher syndrome in Italy.

**Methods:**

Molecular data have been obtained on 75 unrelated Italian patients using the most up-to date technology available for the screening of Usher syndrome gene mutations, i.e., the genotyping microarray developed by Asper Biotech (Tartu, Estonia), which simultaneously investigates 612 different marker positions using the well established arrayed primer extension methodology (APEX).

**Results:**

Using this method, we found that 12% of cases (9 out of 75) harbored homozygous or compound heterozygous mutations in the gene positions analyzed, whereas 20% (15 out of 75) of the patients were characterized by the presence of only one mutated allele based on the positions analyzed. One patient was found to be compound heterozygous for mutations in two different genes and this represents an example of possible digenic inheritance in Usher syndrome. A total of 66.6% of cases (50 out of 75) were found to be completely negative for the presence of Usher syndrome gene mutations in the detected positions. Mutations detected by the array were confirmed by direct sequencing.

**Conclusions:**

These findings highlight the efficacy of the APEX-based genotyping approach in the molecular assessment of Usher patients, suggesting the presence of alleles not yet identified and/or the involvement of additional putative genes that may account for the pathogenesis of Usher syndrome.

## Introduction

Usher syndrome represents a group of recessively inherited disorders characterized by deafness and vision loss and, sometimes, by vestibular areflexia. Usher syndrome is traditionally subdivided into three clinical subclasses (type 1, type 2, and type 3). The subtypes are differentiated by the severity and progression of hearing loss and by the presence or absence of vestibular symptoms, with visual impairment resulting from retinitis pigmentosa (RP) [1] being common to all three subtypes. Usher syndrome shows significant genetic and clinical heterogeneity, and at least 12 distinct loci and 9 genes have been identiﬁed. Although still used in clinical settings, the above mentioned Usher syndrome classification has been challenged by the recent description of many atypical clinical types. Usher syndrome type 1 patients are characterized by the presence of congenital, profound deafness, vestibular dysfunction, and prepubertal onset of progressive RP [2,3]. Usher syndrome type 1 is the most severe form of Usher syndrome. This form accounts for 30%–40% of Usher syndrome cases [3-5]. To date, seven genetic loci for Usher syndrome type 1 (USH1B–H) have been mapped to chromosomes 11q13.5, 11p15.1, 10q22.1, 21q21, 10q21-q22, 17q24-q25, and15q22-q23. Five of the corresponding genes have been identified: 1) the actin-based motor protein myosin VIIa (*MYO7A*, *USH1B*) [6,7], the most common Usher syndrome type 1 genetic subtype, which accounts for 30%–50% of Usher syndrome type 1 cases in the UK and the USA [8,9]; 2) two cadherin-related proteins, namely otocadherin or cadherin 23 (*CDH23*, *USH1D*) [10,11], and protocadherin 15 (*PCDH15*, *USH1F*) [12,13], respectively, representing the second and third most frequent causes of Usher syndrome type 1, and accounting for 10%–35% (*CDH23*) [3-5] and 11% (*PCDH15*) of Usher syndrome type 1 cases in both the UK and USA; 3) the last two Usher syndrome type 1 genes, which encode two scaffold proteins, harmonin (*USH1C*) [1,14] and sans (*USH1G*) [15].

Usher syndrome type 2 is less severe than type 1 and is characterized by congenital moderate-to- severe deafness, with a high-frequency sloping configuration. Owing to an overlap in the clinical appearance of the visual symptoms in types 1 and 2, due to considerable variation in the age of onset, these symptoms are not considered reliable predictors of the type of Usher syndrome in individual cases [2,3,16]. Three genetic loci have been reported so far in Usher syndrome type 2 (*USH2A*, *USH2C,* and *USH2D*). The corresponding genes have been identified. Mutations in the *USH2A* gene on chromosome 1q41, which encode usherin, are the most common mutations and account for up to 85% of Usher syndrome type 2 cases [17,18]. The protein encoded by the *VLGRI* [19] gene at the *USH2C* locus is a member of the serpentine G-protein coupled receptor superfamily. Defects in the Whirlin gene, a PDZ (post-synaptic density, disc-large, Zo-1 protein domains) domain-containing scaffold protein, are responsible for both Usher syndrome type 2 and nonsyndromic hearing loss (DFNB31) [20]. Mutations in *USH2C* and *USH2D* are rare [19,21].

Usher syndrome type 3 is characterized by variable onset of progressive hearing loss, variable onset of RP, and variable impairment of vestibular function [22]. Usher syndrome type 3 is caused by mutations in the *USH3A* (clarin-1) gene, mapped to 3q21-q25 [23,24]. Usher syndrome type 3 is not as common as Usher syndrome type 1 and Usher syndrome type 2, with a prevalence of 2%–4% in all Usher syndrome cases.

Comprehensive molecular diagnostics for Usher syndrome has been hampered both by genetic heterogeneity and the large number of exons for most of the Usher genes. The five Usher syndrome type 1 genes collectively contain 179 protein-coding exons, the two Usher syndrome type 2 genes comprise 162 protein-coding exons, and the Usher syndrome type 3 gene *USH3A* has six protein-coding exons, some of which are alternatively spliced (UCSC Human Genome Browser). Thus far, large-scale mutation screening has been performed using single-strand conformation analysis and denaturing gradient gel electrophoresis, with the subsequent sequence analysis of fragments displaying an aberrant migration pattern. For routine analysis, these techniques are both time consuming and expensive. Microarray technology previously used for mutation analysis of the ATP-binding cassette, sub-family A (ABC1), member 4 (*ABCA4*) gene in patients with either autosomal recessive Stargardt disease or autosomal recessive cone-rod dystrophy, as well as for more than ten genes implicated in Leber congenital amaurosis (LCA), can also be a useful tool for the identification of mutations in patients with Usher syndrome. Inherent to the arrayed primer extension (APEX) is that it only detects established mutations and, its efficiency is, therefore, highly dependent on the extent of earlier mutation analysis efforts. We report on molecular epidemiology data obtained by analyzing the largest set of Italian Usher cases (75 patients) ever collected, using the Usher microarray developed by Asper Biotech, which detects 612 mutations of the nine genes involved.

## Methods

### Sample selection

A total of 75 patients with Usher Syndrome recruited from several Italian centers (Medical Genetics, IRCCS Burlo Garofolo-Children Hospital, Trieste, Italy; Audiology, University of Ferrara, Ferrara, Italy; Department of Ophthalmology of the Second University of Naples, Napoli, Italy); among these, 7 patients were Usher syndrome type 1, 39 were Usher syndrome type 2, and 29 could not be classified. The patients underwent a general screening visit that included a basic ophthalmic consultation, central visual acuity (CVA), Goldman visual field (GVF), fundus oculi, standard electroretinography (ERG), and a series of periodic control visits. The patients also provided a detailed medical history and underwent genetic counseling to identify hereditary patterns. Each patient underwent a complete ear, nose and throat (ENT) and audio-vestibular examination, including otomicroscopic examination, audiometry, and an auditory brainstem response (ABR) for threshold assessment of patients less than 5 years of age. The degree of hearing loss was evaluated for frequencies 0.25–8,000 Hz. We defined mild hearing loss as a loss of 45 dB, moderate loss as a loss of 46–70 dB, severe loss as a loss of 71–90 dB, and profound hearing loss as a loss >90 dB, according to American speech-language-hearing association (ASHA) protocols. Audioprofiles and their median audiograms were used to examine audiological features. A vestibular test was performed on all patients; evaluation of spontaneous and evoked eye movements (nystagmus) and a caloric test (Fitzgerald-Hallpike) were performed under electronystagmograpic registration. All patients underwent neuro-imaging with high-resolution MRI to exclude brain and inner ear malformations. All clinical data was collected in a patient database and was periodically updated at control visits. This study was approved by the ethics committee, followed the Tenets of the Declaration of Helsinki and written consent for genetic testing was obtained from adult probands or parents of minor patients.

### Molecular analysis

Each patient’s genomic DNA, extracted from peripheral blood according to standard protocols, was PCR amplified; amplicons from nine Usher genes were used in the primer extension reaction (APEX) on the Usher genotyping microarray [25-27]. The test on that chip was intended to screen 612 mutations in the genes *MYO7A*, Harmonin, *CDH23*, *PCDH15*, *SANS*, *USH2A*, *VLGR1*, Whirlin, and *USH3A*. These genes involve mutations in patients with *USH1B*, *USH1C*, *USH1D*, *USH1F*, *USH1G*, *USH2A*, *USH2C*, *USH2D*, and *USH3A* subtypes. Mutations included in the chip have previously been described in the literature on Usher syndrome, and they largely refer to Caucasian populations. Microarray containing hundreds of DNA markers in an ordered array allows the simultaneous analysis of these genetic markers. APEX is a resequencing method for identification of established mutations. Oligonucleotide microarrays were designed for each established mutation, with the 5′ end immobilized on the glass slide and the 3′ end immediately adjacent to the variable site. The DNA chips were imaged with the Genorama™ QuattroImager (Asper Biotech, Tartu, Estonia), measuring the fluorescence intensity for each spot, and the sequence variants identified by specific software [28-30]. Each probe lies in a specific position on the DNA-chip; the fluorescence signal corresponding to each probe is specific for the wild-type or mutant nucleotide. Each mutation/polymorphism was identified on an Usher microarray by two unique oligomers, which was specifically designed according to the wild-type sequence of the Usher genes.

### Sanger sequencing

To confirm the results, array-identified genetic variants were validated by direct sequencing, performed in the two centers enrolled in the study (Trieste and Napoli, Italy), using standard protocols. The fragments were resolved on an ABI PRISM® 3700 Genetic Analyzer (Applied Biosystems, Carlsbad, CA), automated sequencer. Wherever possible, segregation of the mutated allele was assessed (in 9 out of 25 positive cases) by Sanger sequencing (see above).

## Results

**Figure 1:**
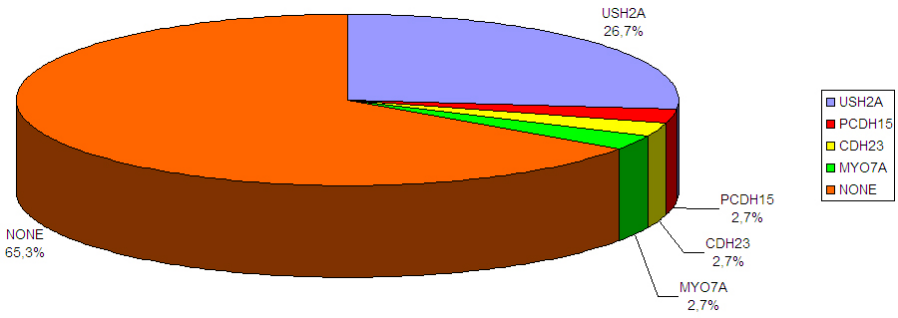
The percentage of positive cases obtained from the molecular screening of the entire series of Italian Usher cases. The chart shows the results of the molecular screening for usherin (*USH2A*); protocaderhin 15 (*PCDH15*); cadherin 23 (*CDH23*); myosin VIIA (*MYO7A*); and the absence of mutated alleles (NONE).

Twenty-five patients with Usher syndrome out of the 75 analyzed (33%) were found to be positive for the presence of pathogenic mutations using the Asper chip. Among these, three patients were positive for the presence of one homozygous mutation, six patients were positive for the presence of two mutations (heterozygous composite), and 15 patients were positive for the presence of one mutated allele, while one patient was positive for the presence of one mutated allele in two different genes, *CDH23* and *PCDH15*. All the remaining cases were completely negative for the presence of mutations (see Figure 1).

The entire detection allele rate was 22.6% (34 pathologic alleles out of 150), and the following genes were positive (see also Table 1) in a decreasing order of relevance: *USH2A* (27 alleles), *CDH23* (3 alleles), *MYO7A* (2 alleles), and *PCDH15* (2 alleles).

**Table 1:** Positive cases detected with the Asper Chip.

**Patient**	**Gene**	**Mutations (first allele)**	**Mutation (second allele)^1^**	**Other non pathogenic variants**	**Clinical details^2^**
101	*USH2A*	p.W3955X (c.11864G>A)	ND		Usher
110	*USH2A*	p.C759F (c.2276G>T)	ND		Usher I
113	*PCDH15*	p.T1867del (c.5601_5603delAAC)	ND		Usher II
115	*USH2A*	p.H308fs (c.921_922insCAGC)	c.1841–2AG splice	p.L555V (c.1663C>G) in *USH2A*	Usher
116	*USH2A*	p.R737X (c.2209C>T)	ND		Usher II
117	*USH2A*	p.E767fs (c.2299delG)	ND	p.T1209A (c.3625A>G) in *CDH23*	Usher II
119	*USH2A*	c.1841–2AG splice	ND	p.L555V (c.1663C>G) in *USH2A*	Usher
120	*USH2A*	p.W3955X (c.11864G>A)	ND	p.R1060W (c.3178C>T) in *CDH23*	Usher
125	*USH2A*	p.Y1123C (c.3368A>G)	ND	p.R302H (c.905G>A) in *MYO7A*	Usher II
129	*USH2A*	p.C536R (c.1606T>C)	ND		Usher II
132	*USH2A*	p.E767fs (c.2299delG)	ND		Usher II
136	*CDH23*	p.T1904T (c.5712G>A)	p.T1904T (c.5712G>A)		Usher I
138	*USH2A*	p.G516V (c.1547G>T)	p.G516V (c.1547G>T)		Usher
141	*USH2A*	p.G713R (c.2137G>C)	p.G713R (c.2137G>C)		Usher
148	*MYO7A*	p.A1340T (c.4018G>A)	ND		Usher II
319	*USH2A*	p.R63X (c.187C>T)	p.W3955X (c.11864G>A)	p.L555V (c.1663C>G) in *USH2A*	Usher
328	*USH2A*	p.A2795S (c.8383G>T)	ND		Usher
331	*USH2A*	p.Y1123C (c.3368A>G)	ND		Usher
334	*USH2A*	p.R626X (c.1876C>T)	p.V1833E (c.5498T>A)		Usher
338	*CDH23*	p.T1209A (c.3625A>G)			Usher
	*PCDH15*		p.T1867del (c.5601_5603delAAC)		
353	*USH2A*	p.R63X (c.187C>T)		p.L555V (c.1663C>G) in *USH2A*	Usher
357	*USH2A*	p.H308fs (c.921_922insCAGC)	p.R626X (c.1876C>T)		Usher
358	*USH2A*	p.G516V (c.1547G>T)	p.G1132D (c.3395G>A)		Usher
389	*MYO7A*	p.R1873W (c.5617C>T)	ND		Usher
395	*USH2A*	p.E767fs (c.2299delG)	ND		Usher

^1^ND: No mutation detected. ^2^Usher I: refers to Usher syndrome type 1; Usher II: refers to Usher syndrome type 2; Usher: refers to Usher syndrome, not classified to any subtype.

The genotyping microarray identified 15 different variants in *USH2A*: of these, the c.1663C>G (p.L555V) variation, previously reported to be a pathogenic variant, is unlikely to represent a mutation, at least in patients 115 and 319 (Table 1). In fact, the c.1663C>G (p.L555V) variation was present in cases in which two clearly pathogenetic mutations were already present; in particular, the c.1663C>G (p.L555V) allele was in *cis* with c.187C>T (p.R63X) nonsense mutation in one case.

Mutation c.3178C>T in the CDH23 gene (patient 120), which corresponded to a protein variation p.R1060W, was classified as “probably non pathologic” by experimental data [31], as well as mutation c.905G>A (p.R302H) in the *MYO7A* gene (patient 125), which has been reported as not being directly responsible for *USH1B* phenotype since it has only a minor effect on the motor activity of myosin VIIa [32].

Finally, mutation c.3625A>G in the *CDH23* gene (patient 117) corresponded to a protein variation p.T1209A, and was predicted as a “benign” non pathogenic allele by PolyPhen2.

According to the clinical classification of patients, pathologic variants were detected in two out of seven patients with Usher syndrome type 1 (28.5%), seven out of 39 patients with Usher syndrome type 2 (18%), and 16 out of 29 patients with unclassified Usher syndrome (55.1%). The mutations in the *USH2A* gene, c.11864G>A (p.W3955X), c.2299delG (p.E767fs), c. 1547G>T (p.G516V) were found with a slightly increased frequency in the Italian population, followed by mutations c.1841–2A>G, c.921_922insCAGC (dupGCCA) (p.H308Fs), c. 3368A>G (p.Y1123C), and c.2137G>C (p.G713R). Moreover, in one case, heterozygous mutations were detected in two different genes, *CDH23* and *PCDH15*; findings compatible with a possible case of digenic inheritance [33]. In this last case, as well as in an additional eight positive cases, segregation analysis was performed to confirm the presence of two independent mutations.

## Discussion

Identification of causal mutations is important for the early diagnosis of Usher syndrome, which is relevant for the decision regarding whether or not to elect for a cochlear implant, for genetic counseling, and prenatal diagnosis. We have described the results of a mutation screening using one of the most up-to-date technologies for a large set of Usher syndrome cases in Italy.

The overall mutation detection rate was 33.3%, including cases positive for the presence of only one mutated allele. This percentage is almost identical to that recently reported for a set of 183 Spanish patients, where the overall mutation detection rate was 33.9% [31]. Our data, together with data obtained in the Spanish study, suggest that the mutation detection rate for the Mediterranean area is probably lower than that reported in other countries when the chip is used [27]. There are several explanations for this finding and they will be discussed later.

One patient was found to be compound heterozygous for mutations in two different genes, one in *PCDH15* and the other in *CDH23*; since proteins interact with one another and form dynamic protein complexes, digenic or oligogenic inheritance of Usher syndrome is not surprising. Indications for digenic inheritance involving mutations in the *PCDH15* and *CDH23* genes have already been obtained in both mice and in human patients [33].

Despite its obvious advantages in terms of rapidity, the current genotyping microarray chip shows some limitations in detecting insertions or deletions; some of the nucleotide variation included in the chip does not have a clearly established pathological implication and needs to be improved; finally, the current chip is only useful for the detection of previously identified mutations.

In conclusion, our data clearly indicate that a) more accurate technologies such as target(s) re-sequencing will be needed for final diagnosis, while genotyping microarray is a robust, low-cost, and rapid technique that is effective for the genetic study of patients with Usher syndrome, b) the array is able to detect variants of an unclear pathologic nature and detection failures have also been observed, c) the results must be confirmed by direct sequencing to avoid misdiagnosis, while continual updates of the microarray should be performed to increase the efficiency and rate of mutation detection. Moreover, the low mutation detection rate in both Italy and Spain [31], might suggest either the presence of many private or population-specific mutations not included in the chip/array, or might refer to the presence of additional putative and as yet, unidentified Usher syndrome genes, which further increases the genetic heterogeneity of this syndrome.
